# Supplementary material for: Duox-generated reactive oxygen species activate ATR/Chk1 to induce G2 arrest in Drosophila tracheoblasts
Source: eLife. 2021 Oct 8;10:e68636. doi: 10.7554/eLife.68636 (PMC8594940; doi:10.7554/eLife.68636)
Supplement: Transparent reporting form [file elife-68636-transrepform1.docx]

***eLife’s*transparent reporting form**

We encourage authors to provide detailed information *within their submission* to facilitate the interpretation and replication of experiments.Authors can upload supporting documentation to indicate the use of appropriate reporting guidelines for health-related research (see [EQUATOR Network](http://www.equator-network.org/%20)), life science research (see the [BioSharing Information Resource](https://biosharing.org/)), or the [ARRIVE guidelines](http://www.plosbiology.org/article/info:doi/10.1371/journal.pbio.1000412) for reporting work involving animal research.Where applicable, authors should refer to any relevant reporting standards documents in this form.

If you have any questions, please consult our Journal Policies and/or contact us:[editorial@elifesciences.org](mailto:editorial@elifesciences.org).

**Sample-size estimation**

- You should state whether an appropriate sample size was computed when the study was being designed
- You should state the statistical method of sample size computation and any required assumptions
- If no explicit power analysis was used, you should describe how you decided what sample (replicate) size (number) to use

Please outline where this information can be found within the submission (e.g., sections or figure legends), or explain why this information doesn’t apply to your submission:

Exact sample sizes of each experiment performed with multiple biological replicates are provided in the corresponding figure legends, supplemental figure legends, or main text as appropriate. Sample size within an experiment was chosen based on number of animals that could be efficiently processed during the procedure, typically n≥2. Final analysis was performed across raw numbers from three biological replicates, thus making the final sample size n≥6.

**Replicates**

- You should report how often each experiment was performed
- You should include a definition of biological versus technical replication
- The data obtained should be provided and sufficient information should be provided to indicate the number of independent biological and/or technical replicates
- If you encountered any outliers, you should describe how these were handled
- Criteria for exclusion/inclusion of data should be clearly stated
- High-throughput sequence data should be uploaded before submission, with a private link for reviewers provided (these are available from both GEO and Array Express)

Please outline where this information can be found within the submission (e.g., sections or figure legends), or explain why this information doesn’t apply to your submission:

Biological replicates were defined as identical repeats of animals/larvae with the same genotype, following the same experimental conditions, whereas technical replicates were animals/larvae of the same genotype within an experimental procedure, typically n≥2. All experiments were performed with multiple biological replicates for each genotype (n≥3) and multiple (n≥2) animals used per replicate.

**Statistical reporting**

- Statistical analysis methods should be described and justified
- Raw data should be presented in figures whenever informative to do so (typically when N per group is less than 10)
- For each experiment, you should identify the statistical tests used, exact values of N, definitions of center, methods of multiple test correction, and dispersion and precision measures (e.g., mean, median, SD, SEM, confidence intervals; and, for the major substantive results, a measure of effect size (e.g., Pearson's r, Cohen's d)
- Report exact p-values wherever possible alongside the summary statistics and 95% confidence intervals. These should be reported for all key questions and not only when the p-value is less than 0.05.

Please outline where this information can be found within the submission (e.g., sections or figure legends), or explain why this information doesn’t apply to your submission:

For comparisons between experimental groups, paired two-tailed Student's t-test was used for estimating significance. Corresponding figure legends include the exact statistical tests used and definition of significance. Raw numerical data included in “Source Data” document.

(For large datasets, or papers with a very large number of statistical tests, you may upload a single table file with tests, Ns, etc., with reference to sections in the manuscript.)

**Group allocation**

- Indicate how samples were allocated into experimental groups (in the case of clinical studies, please specify allocation to treatment method); if randomization was used, please also state if restricted randomization was applied
- Indicate if masking was used during group allocation, data collection and/or data analysis

This does not apply to our submission due to the nature of work presented. This is not a clinical study and all samples within each experiment were separated based on genotype markers.

Please outline where this information can be found within the submission (e.g., sections or figure legends), or explain why this information doesn’t apply to your submission:

**Additional data files (“source data”)**

- We encourage you to upload relevant additional data files, such as numerical data that are represented as a graph in a figure, or as a summary table
- Where provided, these should be in the most useful format, and they can be uploaded as “Source data” files linked to a main figure or table
- Include model definition files including the full list of parameters used
- Include code used for data analysis (e.g., R, MatLab)
- Avoid stating that data files are “available upon request”

Please indicate the figures or tables for which source data files have been provided:

Fig 1L: Numerical data for number of cells in Tr2 DT of wild type, *Btl-SOD1,* and *Btl-Chk1^RNAi^* larvae at different stages.

Fig 1M: Numerical data for mitotic indices in Tr2 DT of wild type and *Btl-SOD1* larvae at different stages.

Fig 2G: Numerical data for number of cells in Tr2 DT of wild type, *Btl-Duox^RNAi^(32903)* and *Btl-Duox^RNAi^(33975),* expressing larvae at different stages.

Fig 2H: Numerical data for mitotic indices in Tr2 DT of wild type, *Btl-Duox^RNAi^(32903)* and *Btl-Duox^RNAi^(33975)* larvae at different stages.

Figure 3D: Numerical data for number of cells in Tr2 DT of wild type, *Btl-Duox^RNAi^(32903)* , *Btl-Duox^RNAi^(32903), Chk1, Btl-Duox^RNAi^(32903), ATR* and *Btl-Duox^RNAi^(32903), Chk1^S373D^* larvae at 16-24 h L3.

Figure 4I: Numerical data for number of cells in Tr2 DT of wild type, *Btl-ATR^RNAi^, Btl-ATRIP^RNAi^, Btl-TOPBP1^RNAi^* and *Btl-Claspin^RNAi^* larvae at L2 and 16-24 h L3.
